# Multimodal Imaging Findings in Penile Squamous Cell Carcinoma

**DOI:** 10.7759/cureus.101081

**Published:** 2026-01-08

**Authors:** Ruben G Ortiz Cordero, Oswaldo A Guevara Tirado, Vinicius Adami Vayego Fornazari, Jinous Saremian, Renato Abu Hana

**Affiliations:** 1 Radiology, University of Florida College of Medicine Jacksonville, Jacksonville, USA; 2 Pathology, University of Florida College of Medicine Jacksonville, Jacksonville, USA

**Keywords:** genitourinary imaging, hpv-associated malignancy, human papillomavirus (hpv), penile lesion, penile squamous cell carcinoma, urethral stenosis

## Abstract

Penile squamous cell carcinoma (SCC) is the most common type of penile malignancy affecting males in their sixth to eighth decade of life. We discuss a case of an 89-year-old male with dementia who initially presented to the emergency department with a penile lesion and symptoms consistent with a urinary tract infection. Further evaluation with MRI and a subsequent biopsy confirmed the diagnosis of penile SCC, which caused multiple strictures in the penile urethra. This report demonstrates a rare instance of penile SCC causing multifocal urethral obstructions and highlights the utility of MRI in characterizing penile lesions.

## Introduction

Penile malignancies account for less than 1% of all male cancers in the United States, with approximately 95% classified as squamous cell carcinoma (SCC) [1-5]. The condition commonly affects males in their sixth to eighth decade of life, with reported five-year survival rates varying widely depending on tumor stage and nodal involvement, ranging from less than 10% in advanced disease to around 90% in early-stage presentations [1-4,6]. Risk factors for the development of penile SCC include viral pathogens (HIV, HPV), inflammatory conditions (balanitis, lichen sclerosus), phimosis, trauma, presence of a foreskin, and poor hygiene [1,3,6]. Clinically, penile SCC presents as a visible or palpable lesion of the glans or prepuce, with symptoms of urinary obstruction typically occurring in more advanced disease [1,2]. In cases with urethral invasion and obstruction, the diagnosis may sometimes be difficult to distinguish from primary urethral carcinoma [1,4].

Given the poor sensitivity of clinical evaluation for detecting invasion of adjacent structures, imaging via ultrasound (US) and MRI is recommended for the assessment of penile cancer [1-7]. Of these two modalities, MRI is preferred because it provides multiplanar imaging and superior soft-tissue contrast [1,2,4-7]. In addition, MRI assists with local staging and treatment planning by identifying tumor extension through the tunica, corporal invasion, urethral involvement, and invasion of surrounding structures [1]. While existing medical literature describes urethral involvement in advanced penile SCC, reported cases more commonly describe focal urethral obstruction rather than multifocal disease [4,8]. This report describes a case of penile SCC causing multifocal urethral obstruction that initially presented as a complicated urinary tract infection (UTI). It also highlights the complementary role of MRI in narrowing the differential diagnosis between penile SCC and primary urethral carcinoma by demonstrating multifocal infiltrative lesions centered in the penile erectile tissues rather than arising from a primary urethral-based mass.

## Case presentation

An 89-year-old male patient with a past medical history of advanced dementia with cognitive impairment and unspecified chronic kidney disease (baseline creatinine of 1.1 mg/dL) presented to the emergency department with increased fatigue, mood instability, aggressiveness, abdominal pain, constipation, and bedwetting for approximately 10 days. Additionally, the patient reported coughing up mucus and consuming less than 10 to 14 ounces of water since symptom onset. He had previously received trimethoprim-sulfamethoxazole for swollen testicles and a suspected UTI, but experienced no improvement. The patient was also reported to have experienced similar symptoms within the past month due to a prior UTI. He denied current dysuria, fever, or chills.

Physical examination was remarkable for a distended, but non-tender abdomen, a mildly edematous scrotum, and a painful, non-bloody, ulcerated-appearing lesion located distally on the ventral surface of the penile shaft and glans. The penis was also uncircumcised and appeared to have a seemingly obliterated urethra. Neither the patient nor the family member could provide further details regarding the lesion’s duration or origin. Vital signs taken on presentation were remarkable for tachycardia (104 bpm) and hypertension (160/69 mmHg). Basic metabolic panel demonstrated an elevated blood urea nitrogen (126 mg/dL) and creatinine (7.61 mg/dL), as well as an estimated glomerular filtration rate of 6. A complete blood count was remarkable only for mild leukocytosis (13.75 x 10⁹/L).

**Figure 1:**
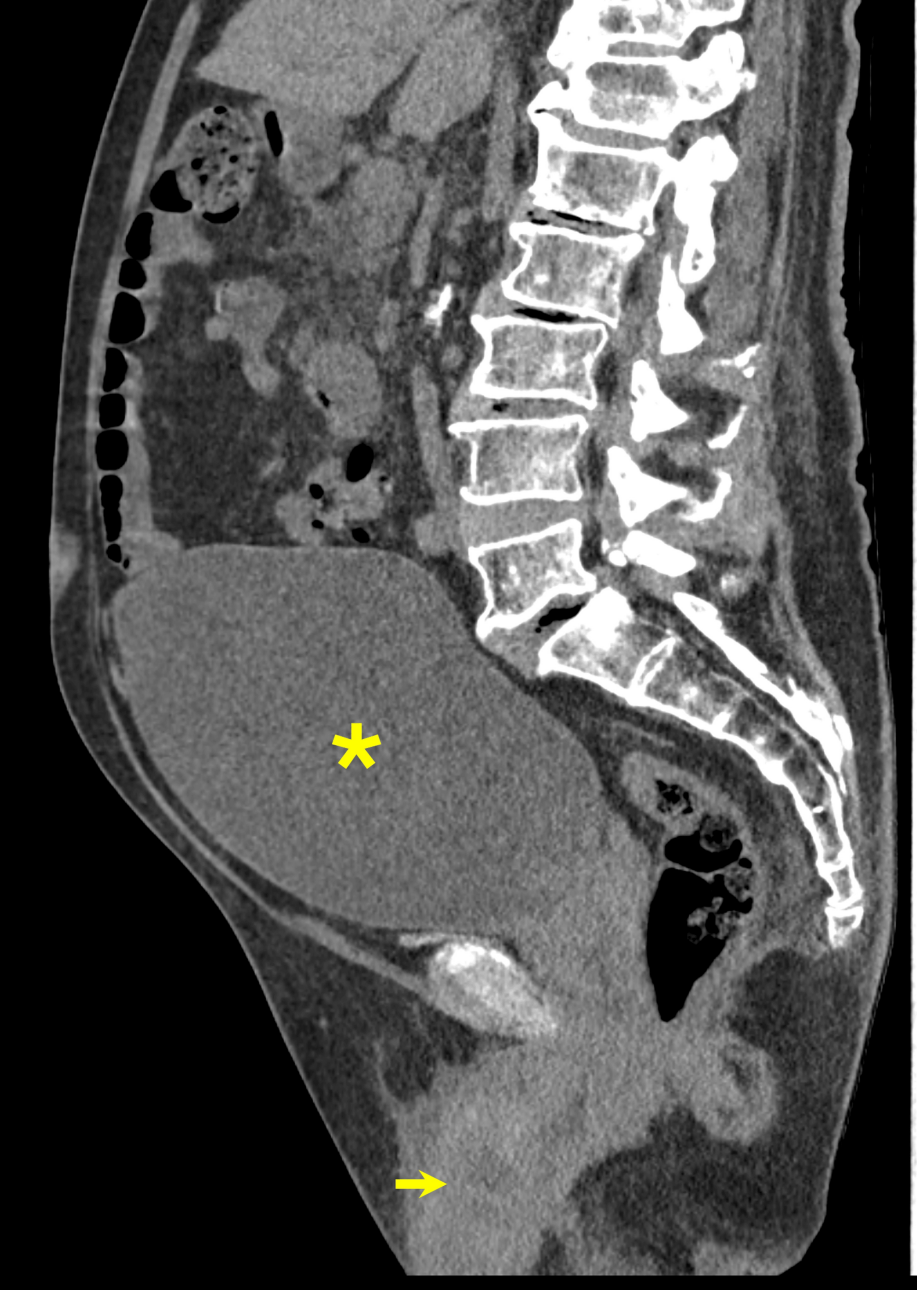
Sagittal non-contrast CT scan of the abdomen and pelvis The image demonstrates a severely distended urinary bladder (asterisk) and multiple irregular penile masses (arrow) CT: computed tomography

A non-contrast CT of the abdomen and pelvis revealed a severely distended urinary bladder with upstream mild to moderate bilateral hydroureteronephrosis (Figure 1). Extensive right periureteral, peripelvic, and perinephric fat stranding was also visualized and was concerning for an ascending UTI. Foley catheter placement was attempted but was ultimately unsuccessful. As a result, urology was consulted, and a suprapubic catheter was placed. Urinalysis obtained from the suprapubic catheter was positive for moderate blood, small leukocyte esterase, and 1+ bacteria and was negative for nitrites. Urine culture obtained from the suprapubic catheter grew >100,000 CFU/mL of Escherichia coli, and both blood cultures and a sexually transmitted infection panel were negative. Bladder decompression via suprapubic catheter resulted in the drainage of >1.5 liters of urine and a net input-output of -2360 mL over the first 24 hours.

Following bladder decompression and appropriate antibiotic administration with piperacillin-tazobactam, the patient’s renal function improved (Table 1). MRI of the abdomen and pelvis (T1-weighted (T1WI), T2-weighted (T2WI), and diffusion weighted imaging (DWI) sequences) with and without contrast (13 mL Dotarem) was subsequently ordered to further characterize the pelvic findings and to evaluate for a potential obstructive or infiltrative etiology not fully assessed on CT.

**Table 1:** Blood urea nitrogen and creatinine levels on admission and following bladder decompression

Lab values	Admission	Post-decompression day 1	Post-decompression day 2	Post-decompression day 3	Units
Blood urea nitrogen	126	117	75.4	56.8	mg/dL
Creatinine	7.61	6.87	3.29	2.46	mg/dL

**Figure 2:**
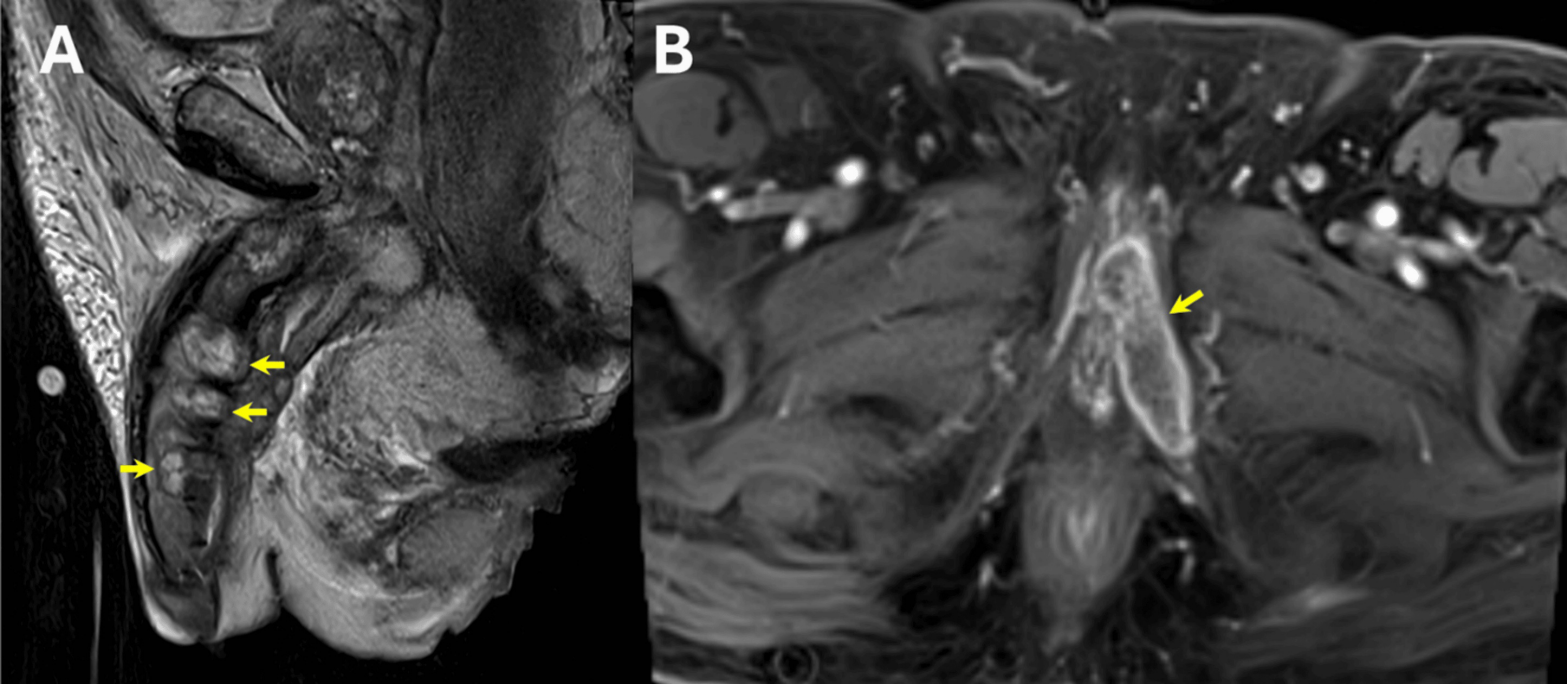
MRI findings (A) Sagittal T2-weighted TSE MRI of the abdomen and pelvis demonstrates multiple irregular mass-like lesions of heterogeneous intensity with central necrosis (arrows) scattered throughout the penis corpus cavernosum and corpus spongiosum extending proximally along the penile shaft and involving the left penile crus. (B) Axial post-contrast T1-weighted MRI with fat suppression showing focal enhancement of the left inferior pubic ramus concerning for osseous spread (arrow) MRI: magnetic resonance imaging; TSI: turbo spin echo

The MRI (3T) of the abdomen and pelvis demonstrated multiple irregular, enhancing, mass-like lesions of heterogeneous intensity with central necrosis scattered throughout the corpora cavernosa and corpus spongiosum of the penis, extending proximally along the penile shaft and involving the left penile crus (Figure 2A). These lesions appeared to be causing multifocal urethral strictures/obliterations, likely preventing successful initial Foley catheter placement. Additionally, there was a disruption of the right tunica albuginea and focal enhancement of the left inferior pubic ramus, radiologically concerning for osseous spread. Nonspecific subcentimeter enhancing inguinal lymph nodes were also seen (Figure 2B). Interventional Radiology was consulted to perform a biopsy.

**Figure 3:**
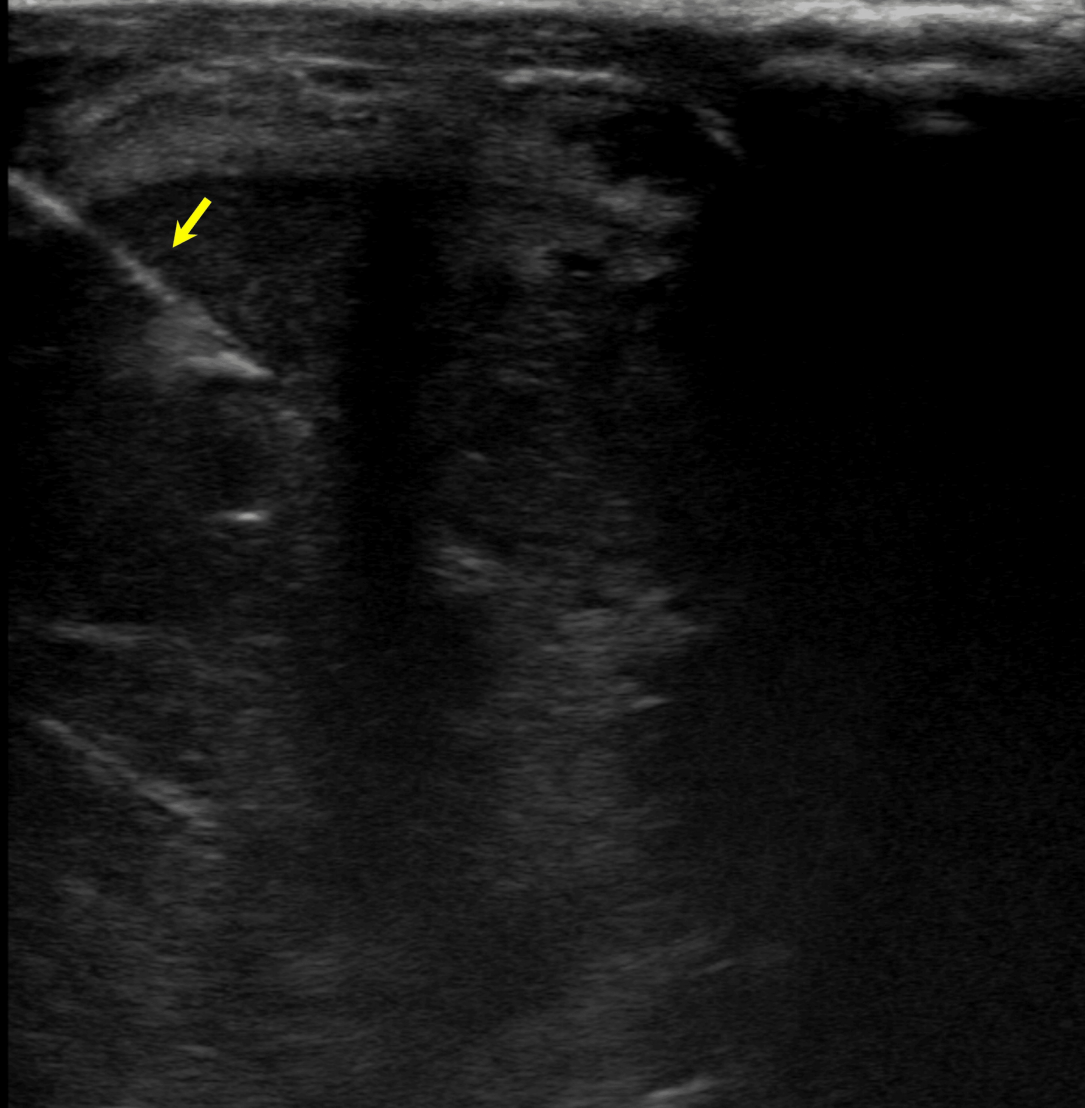
Ultrasound demonstrating 18-gauge BioPince needle (arrow) positioned in the penile lesion for core biopsy

**Figure 4:**
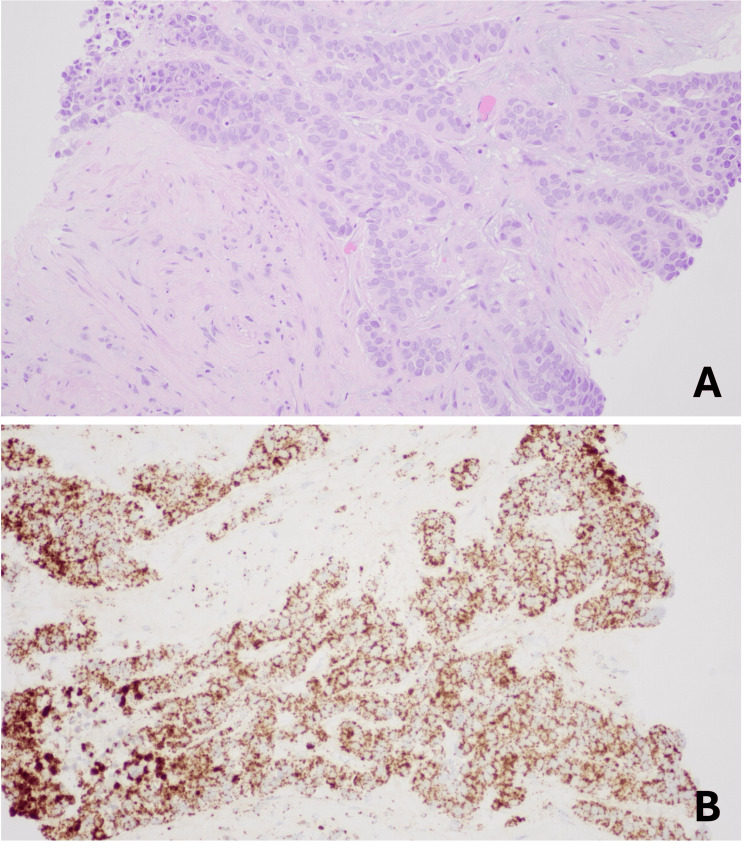
Biopsy findings (A) High-power (X40) hematoxylin and eosin-stained section showing invasive carcinoma with squamoid features, characterized by nests and cords of atypical epithelial cells with eosinophilic cytoplasm, enlarged hyperchromatic nuclei, and irregular nuclear contours within a desmoplastic stromal background. (B) High-risk HPV cocktail in situ hybridization (X20) demonstrates positive nuclear signals in tumor cells, with results consistent with HPV-associated squamous cell carcinoma HPV: human papillomavirus

After obtaining informed consent, the patient was placed in a supine position on the bed. Ultrasound localization of the lesion at the base of the penis within the corpus cavernosum was performed. The site was marked, and lidocaine was injected into the skin and underlying subcutaneous tissues under direct ultrasound visualization. Under ultrasound guidance, an 18-gauge BioPince needle was positioned in the penile lesion (Figure 3). A total of two core biopsy specimens were obtained. The needle was removed, hemostasis was achieved, and sterile dressings were applied. The patient tolerated the procedure well without any immediate complications. Biopsy results were positive for HPV-associated SCC (Figure 4).

Given the patient's advanced age and overall poor clinical condition, he was deemed not suitable for systemic therapy. Following a discussion with the patient’s son, the patient was transitioned to hospice care.

## Discussion

Penile SCC is a rare malignancy that is strongly associated with HIV, HPV, poor hygiene, and phimosis [1,3,4,6]. It typically presents in older male patients as a solitary erythematous, ulcerated, indurated, or bleeding penile lesion on the glans, prepuce, coronal sulcus, or shaft [1,2,6,8]. While urethral involvement can occur in advanced penile SCC, most of the available medical literature appears to focus on localized invasion or a single-site obstruction rather than multifocal disease [4,8,9]. This is because tumors originating from the squamous epithelium of the glans and distal shaft tend to extend contiguously into the anterior urethra [1,4,7,9]. Consequently, when lesions are present in the posterior urethra, they are more likely to represent a primary urethral carcinoma [1,4,7,9]. This report presents an instance where multifocal urethral obstructions were observed in a patient with penile SCC, contributing to an initial clinical presentation of a complicated UTI.

Given that advanced penile SCC can potentially cause obstruction of the urinary tract and contribute to a clinical presentation of a UTI, it is important to discuss the role of imaging to achieve an accurate diagnosis. The most common initial imaging modality for patients presenting with a penile lesion is ultrasonography, as it allows for quick assessment of a lesion’s size and echotexture. Furthermore, the US allows for accurate visualization of the different penile tissue planes, including the tunica albuginea, corpus cavernosum, corpus spongiosum, and urethra [7].

Color Doppler can also reveal changes in lymph node vascularity, providing adjunctive prognostic information as increased peripheral vascularity on a lymph node can be suspicious for metastasis [7]. It is important to know that penile SCC often demonstrates a heterogeneous, hypoechoic texture on US [2,7]. However, a study by Agrawal et al. revealed mixed results on 59 patients with penile cancer, demonstrating that appearance on US can be variable [10]. These results demonstrate that despite the diagnostic utility of US, limitations such as a narrow field of view, operator dependence, and unreliability in delineating invasion at the glans warrant the use of other imaging modalities for a complete evaluation of penile malignancies [5,7,10]. While CT and positron emission tomography (PET) can evaluate lymph nodes and assess for distant disease, they are generally not utilized for local evaluation of penile tumors, as they lack adequate visualization and differentiation of the penile soft tissues [1,2,7]. This was further confirmed in our case, as CT imaging suggested a complicated UTI and did not accurately characterize the underlying malignancy.

Due to its superiority in evaluating soft tissue, MRI is the preferred modality for the evaluation of penile tumors and local spread [7]. However, to maximize its diagnostic capabilities, a proper understanding of normal penile anatomy on MRI is necessary. Normally, the corpus spongiosum and corpus cavernosa demonstrate high signal intensity on T2WI, while the surrounding tunica albuginea and urethral lumen remain hypointense on T2WI [1]. In our case, MRI imaging allowed visualization of several irregular, enhancing, mass-like lesions with heterogeneous intensity and central necrosis throughout the corpus cavernosa and corpus spongiosum, resulting in urethral obstruction at multiple points. Identification of this multifocal urethral stenosis on imaging clarified why the patient presented with bladder outlet obstruction, failed Foley catheter placement, and required placement of a suprapubic catheter. Ultimately, the MRI’s ability to clearly visualize and differentiate soft tissue findings led to the diagnosis of malignancy.

## Conclusions

The significance of this case report lies in describing a unique presentation of advanced penile SCC, in which the tumor caused multifocal urethral obstruction and strictures, as well as emphasizing the importance of multimodal imaging in diagnosis. We also compared the diagnostic utility of US, CT, and MRI and provided imaging descriptions of penile SCC across these modalities. Clinicians and radiologists should maintain a high degree of suspicion for an underlying genital malignancy when encountering a male patient who presents with a penile lesion and recurrent or unexplained obstructive uropathy. Targeted MRI, along with initial US, should be considered for comprehensive anatomical and soft tissue evaluation when imaging findings are not sufficiently explained by infection alone.
